# Platelet-to-lymphocyte ratio and C-reactive protein as markers for colorectal polyp histological type

**DOI:** 10.1186/s12885-021-08221-9

**Published:** 2021-05-17

**Authors:** Rui Chen, Liguang Wang, Qi Zhao, Zhen Li, Man Chen, Guodong Lian, Junyong Zhang

**Affiliations:** 1 Department of Gastroenterology, Shandong Provincial Hospital Affiliated to Shandong First Medical University & Shandong Academy of Medical Sciences, 324 Jingwu Road, Jinan, 250021 Shandong People’s Republic of China; 2 Department of Oncology, Shandong Provincial Hospital Affiliated to Shandong First Medical University & Shandong Academy of Medical Sciences, Jinan, Shandong China; 3 Department of Critical Care Medicine, Shandong Provincial Hospital Affiliated to Shandong First Medical University & Shandong Academy of Medical Sciences, Jinan, Shandong China; 4 Department of Gastrointestinal Surgery, Shandong Provincial Hospital Affiliated to Shandong First Medical University & Shandong Academy of Medical Sciences, Jinan, Shandong China

**Keywords:** Platelet-to-lymphocyte ratio (PLR), C-reactive protein (CRP), Colorectal polyps, Histological type

## Abstract

**Background:**

The platelet-to-lymphocyte ratio (PLR) and C-reactive protein (CRP) level are markers that have been reported to predict the histological type of various tumors, and here, we evaluated their utility in predicting colorectal polyp histological types.

**Methods:**

We retrospectively reviewed 172 patients with colorectal polyps who underwent endoscopic polypectomy. The associations between histological type and clinicopathologic parameters were assessed by multivariate analysis.

**Results:**

The optimal PLR and CRP cut-off values were 113.32 and 0.39, respectively. The PLR (*P* = 0.002) and CRP (*P* = 0.009) values were associated with the histological type according to the univariate analysis, whereas low PLR (*P* ≤ 0.001) and CRP (*P* = 0.017) values were independent risk factors in the multivariate analysis together with maximum tumor diameter (*P* ≤ 0.001) and tumor number (*P* = 0.0014).

**Conclusions:**

Preoperative PLR and CRP are correlated with the colorectal polyp histological type.

## Plain language summary

Previously, the platelet-to-lymphocyte ratio (PLR) and C-reactive protein (CRP), which are two inflammation-related biomarkers, were confirmed to be prognostic indicators in early gastric cancer, gastrointestinal cancers and CRC.

However, to our knowledge, no studies have reported the association among PLR, CRP and histological type of colorectal polyps.

Further multivariate logistic regression analysis revealed that a maximum tumor diameter > 1.35 cm (odds ratio [OR] = 9.277, *P* < 0.001), number of tumors (OR = 2.807, *P* = 0.014), a CRP level > 0.39 (OR = 0.262, *P* = 0.017) and a PLR > 113.32 (OR = 0.210, *P* < 0.001) were independent variables for predicting the histological type of colorectal polyps, whereas location and physician were not Table 3.

We found that both the PLR and CRP are novel preoperative biomarkers that are closely related to the histopathological type of colorectal polyps. In our study, the PLR and CRP were shown to be economical, reliable, and convenient tools for predicting the histopathological type of colorectal polyps.

## Background

Colorectal cancer (CRC) is one of the most common malignancies of the digestive tract, and its mortality rate in men and women has increased so that it now ranks third in terms of cancer-related deaths among global malignancies [1]. Colonoscopy is the gold standard for CRC diagnosis. Most colorectal CRCs develop through the well-known adenoma-carcinoma sequence [2] and arise from precancerous polyps (adenomas or serrated polyps), which is a process that requires years [3]. By detecting and removing adenomatous polyps, the incidence and mortality of CRC can be reduced. Some evidence suggests that for every 1.0% increase in adenoma detection, the risk of colorectal cancer is decreased by 3.0%. The risk that polyps will develop into cancer depends on their histological type (villous or tubular) [4]. Studies have shown that the surgical resection of colorectal polyps, such as adenomas, which are prone to malignant transformation, can help reduce the incidence and mortality rates of CRC [5].

Regarding the assessment of the histological type of colorectal polyps, the number of cases of colorectal polyps that are diagnosed and treated by colonoscopy has now increased, and the pathological type is subsequently diagnosed by an experienced pathologist. However, considering economic efficiency and feasibility, colonoscopy techniques are difficult to use extensively in the clinic. Therefore, an effective biomarker that can be conveniently implemented for widespread use for the prediction of the histological type in patients with colorectal polyps is urgently needed.

Recently, many studies have shown that the systemic inflammatory response is related to tumor progression. The platelet-to-lymphocyte ratio (PLR) and C-reactive protein (CRP), which are two inflammation-related biomarkers, have been confirmed to be prognostic indicators in early gastric cancer, gastrointestinal cancers, and CRC [6–8]. However, to our knowledge, no studies have reported the association among PLR, CRP, and the histological type of colorectal polyps.

Therefore, this study was designed to assess the relationships among PLR, CRP and colorectal polyp histological type and to identify simple and practical inflammation-related variables and clinicopathological factors that can be used to predict the histological type of colorectal polyps.

## Methods

### Patients

We retrospectively evaluated the data of 172 patients with colorectal polyps who underwent endoscopic polypectomy and who were diagnosed by postoperative pathologic analysis from July 2018 to September 2019 at Shandong Provincial Hospital affiliated with Shandong First Medical University, China. The exclusion criteria for this study were (1) no pathological diagnosise of the polyp, (2) severe infection, (3) hematopathy, and (4) malignancy. Most patients were excluded due to a lack of complete case data. Eventually, 172 patients with colorectal polyps were included in this study. The clinicopathological data, including sex, age, maximum tumor diameter, location, physician, number of tumors, smoking status, and drinking status, were collected by two reviewers who did not participate in the data analysis.

This study was approved by the Ethics Committee of the Shandong Provincial Hospital affiliated with Shandong First Medical University. All patients provided written informed consent.

### Endoscopic polypectomy procedures

Endoscopic resection (ER), including endoscopic polypectomy (EP), endoscopic mucosal resection (EMR) and endoscopic submucosaldissection (ESD) are used to remove superficial neoplasms from the colon. Endoscopic submucosal dissection (ESD), has been introduced to remove the larger colorectal polyps. EMR and ESD require submucosal injections than conventional snares polypectomy or biopsy techniques. EMR requires the removal of the diseased mucosa with a high frequency trap, and the specimens recovered from the basket are sent for pathological examination. ESD needs to be labeled around the lesion and complete dissection is performed with a scalpel.

### Peripheral blood sample analysis

All patients underwent a preoperative hematology examination. All patients fasted for 6–8 h prior to the blood draw. Blood samples were collected in ethylenediaminetetraacetic acid tubes within 7 days before surgery (median time, 3.1 days). Routine blood tests were performed using an automated hematology analyzer (Sysmex XN-9000; Sysmex Co. Kobe, Japan) and included neutrophil, lymphocyte, and platelet counts. Liver function tests were performed using a different automated hematology analyzer (Beckman Coulter AU5800; Beckman Coulter Co, Japan) and included measurements of the CRP levels.

### Definition and grouping of PLR and CRP

The PLR is calculated as the platelet count divided by the lymphocyte count. Since PLR and CRP have not yet been reported to be associated with the histological type of colorectal polyps, a receiver operating characteristic (ROC) curve was used in this study to determine the most appropriate cut-off values [9]. The value with the most approximate index was selected, and the patients were then divided into two groups based on cut-off value.

Continuous variables are expressed as the mean ± standard deviation and the median (minimum: maximum). The box plot shows the distribution of PLR and CRP in the tubular adenoma group and the tubulovillous adenoma group. The χ2 test was used for the univariate analysis. To determine independent risk factors, a multivariate logistic regression analysis was performed for variables for which *P* < 0.05 (two-tailed) was obtained in the univariate analysis. *P* < 0.05 (two-tailed) was considered statistically significant, and all statistical analyses were performed by SPSS software (version 21.0; Chicago, IL).

## Results

### Clinicopathological characteristics

Of the 172 patients with colorectal polyps,130 patients (75.6%) were men and 42 (24.4%) were women; their mean age was 55.4 ± 10.5 years. Postoperative pathological analysis confirmed tubular adenoma in 115 patients (66.9%) and tubulovillous adenoma in 57 patients (33.1%). Other clinicopathological characteristics, including sex, age, maximum tumor diameter, location, physician, number of tumors, smoking status, and drinking status, are shown in Table 1. The characteristics of the validation group are also shown in Table 1.

**Table 1:** Patient demographics (*N* = 172)

Variable	N (%)
Age (year), mean ± SD	55.4 ± 10.5
Sex	
Male	130(75.6)
Female	42(24.4)
Location	
Colon	129(75)
Rectum	43(25)
Histological type	
Tubular adenoma	115(66.9)
Tubulovillous adenoma	57(33.1)
Maximum tumor diameter (cm), median (range)	1.0(0.3–7.0)
Smoking	
Yes	63(36.6)
No	109(63.4)
Drinking	
Yes	88(51.2)
No	84(48.8)
Physician	
Experienced	107(62.2)
Unexperienced	65(37.8)
Number of tumors, mean ± SD	4.7 ± 8.5

### Relationship between inflammation-based variables and histological type

**Fig. 1:**
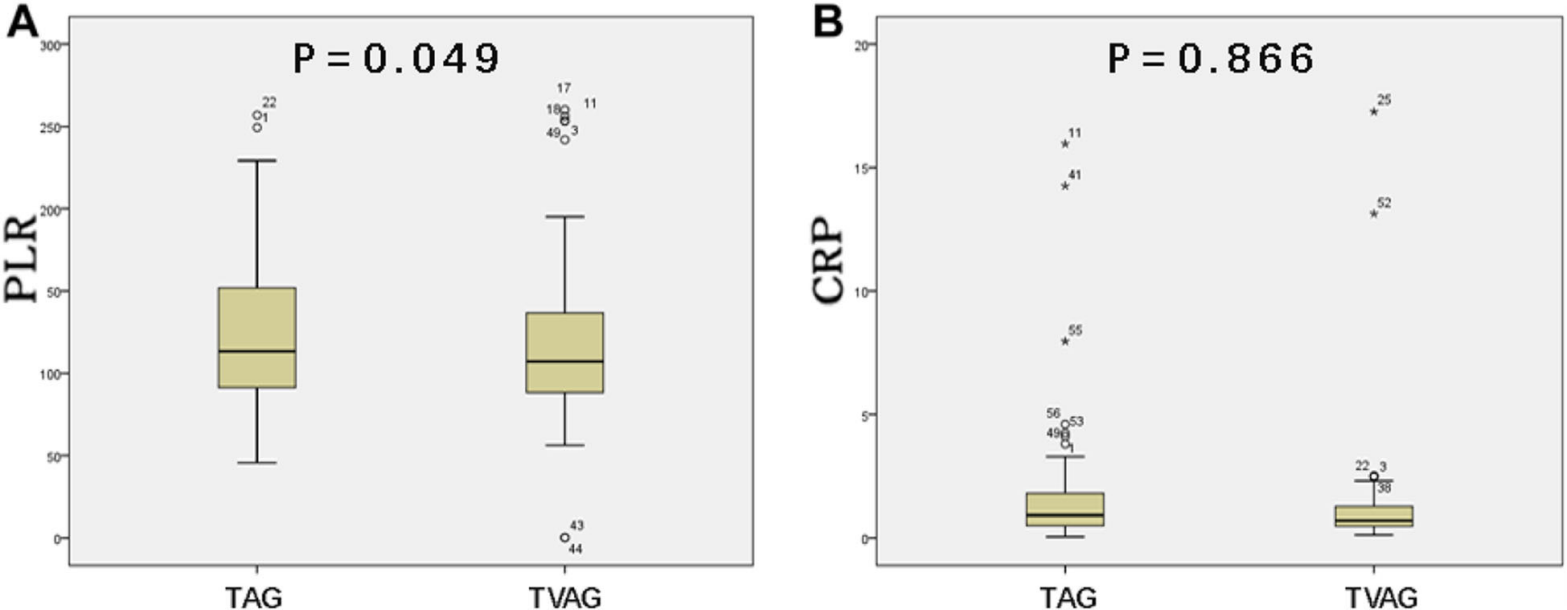
Distribution of PLR and CRP between the tubular adenoma group (TAG) and the tubulovillous adenoma group (TVAG). **a** The PLR value in the tubular adenoma group was higher than that in the tubulovillous adenoma group (*P* = 0.049). **b** The value of CRP in the tubular adenoma group was higher than that in the tubulovillous adenoma group (*P* = 0.866)

The PLR and CRP values were significantly higher in the tubular adenoma group than in the tubulovillous adenoma group (Fig. 1).

### The optimal thresholds for PLR and CRP

**Fig. 2:**
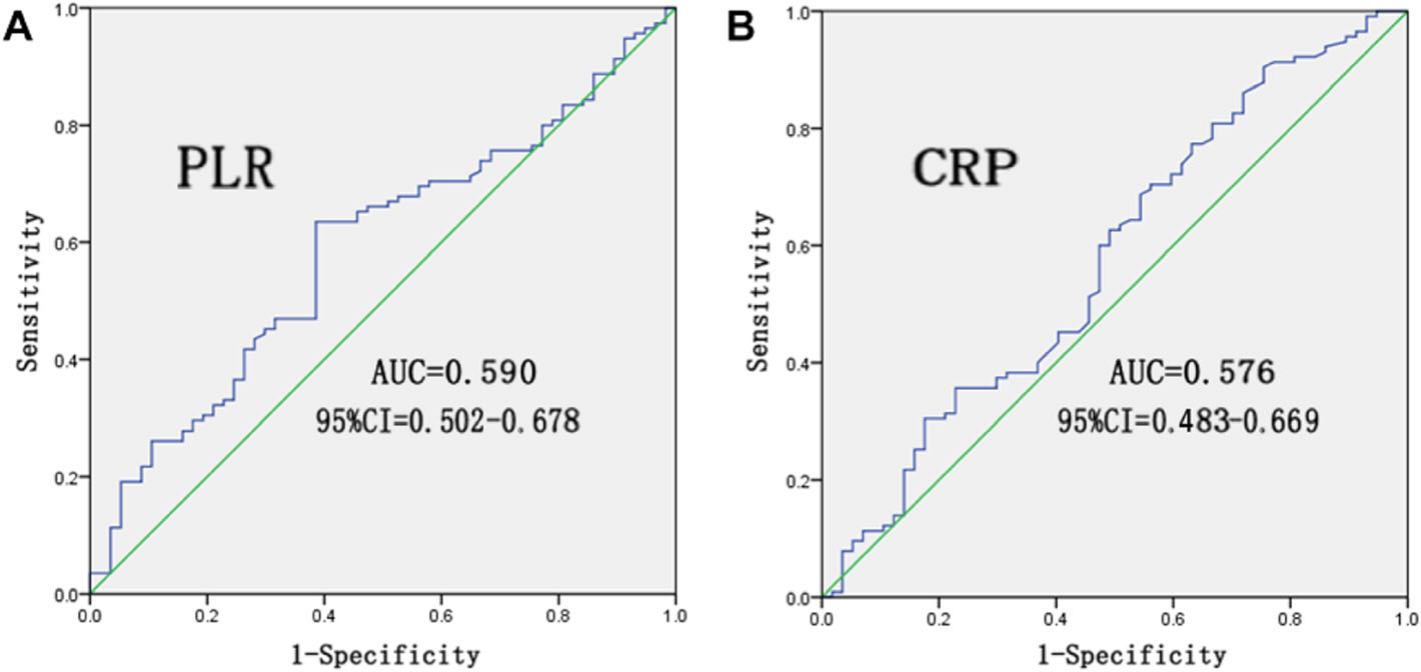
ROC curve for PLR and CRP in the tubular adenoma group and tubulovillous adenoma group. **a** AUC for PLR: 0.590; 95% CI: 0.520–0.678. **b** AUC for CRP: 0.576; 95% CI: 0.483–0.669

According to the ROC curves, the area under the curve (AUC) values of the PLR and CRP were 0.590 (95% confidence interval (CI): 0.502–0.678) and 0.576 (95% CI: 0.483–0.669), respectively, and the corresponding optimal cut-off values for the PLR and CRP were 113.32 and 0.39, respectively (Fig. 2). Patients with colorectal polyps were then separated into a high PLR group (> 113.32) and a low PLR group (< 113.32) as well as a high CRP group (> 0.39) and a low CRP group (< 0.39). The numbers of patients in the high PLR and high CRP groups were 73 (63.5%) and 104 (90.4%), respectively.

### Univariate and multivariate analyses of factors associated with the histological type of colorectal polyps

A univariate analysis showed that high preoperative PLR (*P* = 0.002, χ2 = 9.542) and CRP (*P* = 0.009, χ2 = 6.899), location (*P* = 0.031, χ2 = 4.627), maximum tumor diameter (*P* < 0.001, χ2 = 33.482), physician (*P* = 0.012, χ2 = 6.347) and number of tumors (*P* = 0.048, χ2 = 3.912) were significantly related to the histological type of colorectal polyps. Nevertheless, no significant associations were observed between histological type and age, sex, smoking, drinking, neutrophil count, lymphocyte count, platelet count, monocyte count, NLR or the lymphocyte-to-monocyte ratio (LMR) (Table 2). Further multivariate logistic regression analysis revealed that a maximum tumor diameter > 1.35 cm (odds ratio [OR] = 9.277, *P* < 0.001), number of tumors (OR = 2.807, *P* = 0.014), a CRP level > 0.39 (OR = 0.262, *P* = 0.017) and a PLR > 113.32 (OR = 0.210, *P* < 0.001) were independent variables that could predict the histological type of colorectal polyps, whereas location and physician were not (Table 3)**.**

**Table 2:** Univariate analysis of clinicopathologic variables according to LNM

Characteristics	Tubular adenoma group (*n* = 115)	Tubulovillous adenoma group (*n* = 57)	Univariate analysis	
			X^2^	*P*
Age (years)			1.318	0.251
≤ 58	73(63.5)	31(54.4)		
> 58	42(36.5)	26(45.6)		
Sex			0.523	0.469
Male	85(73.9)	45(78.9)		
Female	30(26.1)	12(21.1)		
Location			4.627	0.031*
Colon	92(80.0)	37(64.9)		
Rectum	23(20.0)	20(35.1)		
Maximum tumor diameter (cm)			33.482	0.000*
≤ 1.35	87(75.7)	17(29.8)		
> 1.35	28(24.3)	40(70.2)		
Smoking			0.936	0.333
Yes	45(39.1)	18(31.6)		
No	70(60.9)	39(68.4)		
Drinking			0.003	0.958
Yes	59(51.3)	29(50.9)		
No	56(48.7)	28(49.1)		
Physician			6.347	0.012*
Experienced	64(55.7)	43(75.4)		
Unexperienced	51(44.3)	14(24.6)		
Number of tumors				0.048*
≤ 4	82(71.3)	32(56.1)		
> 4	33(28.7)	25(43.9)	3.921	
Neutrophil count(× 10^9^/L)			2.358	0.125
Low	82(71.3)	34(59.6)		
High	33(28.7)	23(40.4)		
Lymphocyte count(×10^9^/L)			0.845	0.358
Low	59(51.3)	25(43.9)		
High	56(48.7)	32(56.1)		
Platelet count(×10^9^/L)			2.988	0.084
Low	65(56.5)	40(70.2)		
High	50(43.5)	17(29.8)		

Continuous variables are expressed as the mean ± standard deviation, and categorical variables are expressed as N (%)

The values given are the number of patients unless indicated otherwise

*Represents *P* < 0.05, which was considered to be statistically significant

**Table 3:** Multivariate analysis to evaluate potential predictive factors for Histologic type in patients

Characteristics	OR(95% CI)	*p*
Location		0.100
Colon	1.000	
Rectum	2.107 (0.868–5.115)	
Maximum tumor diameter (cm)		< 0.001*
≤ 1.35	1.000	
>1.35	9.277(3.730–23.069)	
Physician		0.545
Experienced	1.000	
Unexperienced	1.328(0.529–3.334)	
Number of tumors		0.014*
≤ 4	1.000	
>4	2.807(1.234–6.384)	
CRP count		0.017*
Low	1.000	
High	0.262(0.087–0.786)	
PLR		< 0.001*
Low	1.000	
High	0.210(0.090–0.493)	

*OR* Odds ratio

*Represent *P* < 0.05, which was considered to be statistically significant

## Discussion

Colorectal polyps are bumpy lesions on the surface of the intestine. In recent years, many studies have found that colorectal polyps are significantly associated with CRC, and the removal of these polyps has been shown to reduce the risk of developing CRC. Adenomatous polyps are currently recognized as precancerous lesions in CRC, and 67.1% of CRCs are derived from adenomatous polyps. The association between adenomas and carcinomas, the incidental discovery of co-benign adenomas in CRCs, and ras mutations and chromosomal changes in benign adenomas and carcinomas also support the adenoma-carcinoma sequence [10]. The course of CRC development occurs roughly through the path of proliferative adenoma-tubular adenoma-villous adenoma-early carcinoma-invasive carcinoma [11]. Since most screening and intervention programs for colon cancer target the elimination of traditional adenomas, the reduction rate of CRCs using our current approach can be as high as 65% [2]. To maintain relatively normal intestinal function and improve the postoperative quality of life, minimally invasive treatment, including endoscopic submucosal dissection (ESD) and endoscopic mucosal resection (EMR), has become a routine approach for the treatment of colorectal polyps [12]. After the resection of colorectal neoplasia, it is recommended that colonoscopy be performed once a year until all colorectal polyps, including small lesions, are completely eliminated and once every 3 years thereafter [13].

Three types of adenomatous polyps have been defined: tubular adenomas, villous adenomas, and tubulovillous adenomas. It is estimated that the average annual conversion rate in patients with adenomas is 0.25% and that the annual conversion rates for large (≥10 mm) adenomas, villous adenomas, and severely stunted adenomas are 3, 17, and 37%, respectively [14]. One study showed that approximately one-third of cancers are derived from adenomatous polyps and that one-third are derived from villous adenomas. Given the much higher incidence of adenomatous polyps, these numbers suggest that villous growth patterns are more likely to lead to malignancy compared with typical adenomatous polyps [11]. By colonoscopy, only a general diagnosis according to the shape of the polyp can be made, whereas complete polyp resection and pathological examination can determine the nature of the polyp. All adenomas are pathologically classified by a pathologist [15], and pathologic analysis is the “gold standard” of diagnosis. Biopsies should not be performed in cases of polypectomy or EMR because of the increased medical costs. However, biopsy of a lesion suspected to be T1 carcinoma may be acceptable because histological information helps decide the appropriate treatment strategies. For superficial lesions (flat or sunken lesions), biopsy should not be performed before endoscopic resection, as false non-presentation may occur after EMR injection due to submucosal fibrosis [16]. Risk, cost, and effectiveness should be considered when discussing different options [17]. The advantage of a full colonoscopy, of course, is that the entire colon can be evaluated while a biopsy or polypectomy is performed. However, colonoscopy and biopsy are costly and time- intensive. Many markers have been investigated including carcinoembryonic antigen (CEA) and carbohydrate antigen 19–9 (CA 19–9). Due to the poor sensitivity and specificity of CEA detection methods, monitoring CEA in CRC provides only a modest improvement in patient prognosis. CA 19–9 level has also been shown to be 80% effective in the detection of pancreatic cancer [18]. No additional biomarkers have been found to be associated with colorectal polyps. Therefore, a new, reliable and economically feasible biomarker is urgently needed to predict the histological types of colorectal polyps.

The link between inflammation and malignancy has been well established since Virchow first proposed it in 1863 [19]. Inflammation causes systemic changes in the tumor microenvironment that facilitate tumor progression. Cancer and inflammation are closely related, and both local and systemic changes in inflammatory parameters are observed in cancer patients [20]. The PLR has been gradually recognized as a new inflammatory marker and an influential factor that affects the prognosis of malignant tumors. Lymphocytes, including subsets such as CD8+ and CD3+ T cells, are associated with a good prognosis in patients with certain tumors [21]. The upregulation of circulating platelets promotes tumor progression through the secretion of growth factors by activated platelets, which protects tumor cells from immune attack, promotes the growth of tumor cells near the endothelium, and enhances tumor proliferation and mobility [22]. With regard to the PLR, previous studies have shown that some platelet receptors, such as GP1b/IX/V and P-selectin, are associated with cancer growth since they promote angiogenesis through expression of cytokines and vascular endothelial growth factor (VEGF) and because they promote tumor progression [23]. CRP is one of the most commonly used indicators in hospital settings to assess the strength of the systemic inflammatory response because of its high sensitivity, good specificity, and high repeatability [24]. CRP plays an important role in the development and/or prognosis of a variety of cancers, including esophageal cancer, hepatocellular carcinoma (HCC), and non-small cell lung cancer [25–27].

Ample evidence supports the prognostic value of PLR and CRP in some solid tumors. Koji et al. [28] recruited 141 patients who underwent curative resection for HCC and reported that the preoperative serum CRP level was an independent and important indicator of poor prognosis and early recurrence in HCC patients. In a retrospective study [26] involving 216 patients with HCC who were treated with resection or nonsurgical treatment, a CRP level > 1 mg/dl was an independent risk factor for HCC recurrence, with a 5-year recurrence rate of 27.4% vs. 16.4% (HR 2.33; 95% CI 1.13–4.83; *P* = 0.022). Lian et al. [29] retrospectively analyzed 162 patients with resectable gastric cancer and divided them into groups according to the median preoperative PLR value (PLR low: < 208 or PLR high: > 208). PLR measurements can provide important diagnostic and prognostic information for patients with resectable gastric cancer. Georgiana et al. [8] retrospectively analyzed 391 patients who were admitted to the hospital and who underwent surgery for complicated CRC. In the multivariate regression analysis, the increase in the PLR resulted in an increased risk of death (hazard ratio (HR) = 1.024; 95% CI = 1.019.1.029; *P* value = 0.000000), and the PLR was thus considered an independent risk factor. However, few articles mention PLR and CRP as predictors of colorectal polyps. A study of 60 patients with colorectal adenomatous polyps showed that serum CRP concentrations were higher in patients with adenomas located proximally (8.674 ± 9.19 μg/ml) compared with the control group (4.94 ± 5.53 microg/ml; *P* < 0.05) and that CRP levels may be associated with the development of tumors in the proximal part of the colon. To date, no study has reported the relationships among PLR, CRP and the histopathological types of colorectal polyps.

In this study, we first discussed the significance of the PLR and CRP levels in their ability to predict the histopathological type of colorectal polyps. The results showed that in the tubular adenoma group, the platelet counts and CRP levels were higher than those in the tubulovillous adenoma group and the lymphocyte counts were lower than those in the tubulovillous adenoma group, but the differences were not statistically significant. Based on this result, we speculate that the detection of these factors in colorectal polyps may not be sensitive enough to reflect these single differences during the early stages of tumor progression. However, the PLR amplifies the differences in the single distribution. Here, we found a significant difference in the PLR distribution between tubular and tubulovillous adenomas. Since no study has assessed the correlation between the PLR or CRP level and the histopathological type of colorectal polyps, ROC curves were used to determine the cut-off values. In the univariate and multivariate analyses, both low PLR and low CRP were associated with the histopathological types, while low PLR and low CRP were shown to be independent risk factors.

Various studies have recommended different cut-off points for the PLR (between 106 and 130) and CRP (approximately 1 mg/ml). In this study, the cut-off point of the PLR was 113.32, while the cut-off point of CRP was 0.39 [4, 6, 26, 30]. Various methods of calculating PLR and CRP, and different, nonstandardized study populations may have contributed to these differences.

This study has several limitations. First, this is a retrospective analysis of patients at a single center. Second, our study was limited to patients whose histopathological types were confirmed as tubular adenoma or tubulovillous adenoma, and we did not analyze patients with other pathological types of colorectal polyps, and thus, our sample size was small. The second limitation was that we assessed only preoperative laboratory values. Further studies are needed to understand the role of the PLR and CRP alone or in combination with other inflammatory biomarkers in predicting the histopathological type of colorectal polyps.

## Conclusions

This study is first to investigate the relationship between inflammatory factors (PLR and CRP) and histopathological type in patients with colorectal polyps. We found that both the PLR and CRP are novel preoperative biomarkers that are closely related to the histopathological types of colorectal polyps. In our study, the PLR and CRP were shown to be economical, reliable, and convenient tools for predicting the pathological type of colorectal polyps.

## Data Availability

Data related to this article can be acquired from the corresponding author.

## Abbreviations

PLR: The platelet-to-lymphocyte to lymphocyte ratio
CRP: C-reactive reactive protein
CRC: Colorectal cancer
AUC: Area under the curve
LMR: The lymphocyte -to-monocyte ratio
ER: Endoscopic resection
EP: Endoscopic polypectomy
ESD: Endoscopic submucosal dissection
EMR: Endoscopic mucosal resection
CEA: Carcinoembryonic antigen
CA 19–9: Carbohydrate antigen 19–9
VEGF: Vascular endothelial growth factor
HCC: Hepatocellular carcinoma
